# Supplementary material for: Development of a novel multi-epitope mRNA vaccine candidate to combat SFTSV pandemic
Source: PLoS Negl Trop Dis. 2025 Jan 22;19(1):e0012815. doi: 10.1371/journal.pntd.0012815 (PMC12788908; doi:10.1371/journal.pntd.0012815)
Supplement: S2 Text — (DOCX) [file pntd.0012815.s002.docx]

tPA sequence (UniProt ID: [P00750](https://www.ncbi.nlm.nih.gov/protein/P00750) )

MDAMKRGLCCVLLLCGAVFVSPS

MITD sequence (UniProt ID: [Q8WV92](https://www.ncbi.nlm.nih.gov/protein/Q8WV92)）

MAKSGLRQDPQSTAAATVLKRAVELDSESRYPQALVCYQEGIDLLLQVLKGTKDNTKRCNLREKISKYMDRAENIKKYLDQEKEDGKYHKQIKIEENATGFSYESLFREYLNETVTEVWIEDPYIRHTHQLYNFLRFCEMLIKRPCKVKTIHLLTSLDEGIEQVQQSRGLQEIEESLRSHGVLLEVQYSSSIHDREIRFNNGWMIKIGRGLDYFKKPQSRFSLGYCDFDLRPCHETTVDIFHKKHTKNI

Kozak sequence

GCCATGATGG
