# Supplementary material for: Development of a novel multi-epitope mRNA vaccine candidate to combat SFTSV pandemic
Source: PLoS Negl Trop Dis. 2025 Jan 22;19(1):e0012815. doi: 10.1371/journal.pntd.0012815 (PMC12788908; doi:10.1371/journal.pntd.0012815)
Supplement: S5 Text — (DOCX) [file pntd.0012815.s005.docx]

RNA:

AUGGACGCCAUGAAGCGCGGCCUGUGCUGCGUGCUGCUGCUGUGCGGCGCCGUGUUCGUGAGCCCCAGCGGCAUCGGCGACCCCGUGACCUGCCUGAAGAGCGGCGCCAUCUGCCACCCCGUGUUCUGCCCCCGCCGCUACAAGCAGAUCGGCACCUGCGGCCUGCCCGGCACCAAGUGCUGCAAGAAGCCCGAGGCCGCCGCCAAGAGCAGCUACUUCGUGCCCGACGCCCGCGCCGCCUACACCAGCGUGGAGGCCGUGGCCAACUACGCCGCCUACAAGCAGGUGUUCCGCAGCCGCACCAAGGCCGCCUACGUGAGCCUGAGCUUCGACCACGCCGUGGCCGCCUACCCCACCUUCGACGGCUACGUGGGCUGGGCCGCCUACAAGCCCAGCGUGUGGUUCCUGCAGGCCGCCGCCUACUACCUGCCCGUGGGCCCCGCCGUGAUGGCCGCCUACAUGGCCUUCGGCAGCCUGAUCCCCACCGCCGCCUACUUCACCAAGACCAUCAACGUGAAGAUGCACGAGUACGGCGCCGAGGCCCUGGAGCGCGCCGGCGCCGCCUGGGUGCCCAGCGCCGUGAUCGAGCUGACCAUGCCCAGCGGCCCCGGCCCCGGCCAGCCCUUCGACGUGGCCUGGAUGGACGUGGGCCACAGCCACAAGGGCCCCGGCCCCGGCAAGAGCACCGAGAUCCACUUCCACAGCGGCAGCCUGGUGGGCAAGGGCCCCGGCCCCGGCAAGAGCACCGAGAUCCAGUUCCACAGCGGCAGCCUGGUGGGCAAGGGCCCCGGCCCCGGCCACAGCCAGUUCCAGGGCUACGUGGGCCAGCGCGGCGGCCGCAGCGGCCCCGGCCCCGGCACCUUCCUGGAGCUGAAGAGCUUCAGCCAGAGCGAGUUCCCCGACGGCCCCGGCCCCGGCCUGAUGGCCCUGCAGGAGAAGUACGGCCUGGUGGAGCGCGCCGAGCACGAGUACGGCGCCGAGGCCCUGGAGCGCGCCGGCAUCCGCGGCAGCUUCAGCGUGAACUACCGCGGCCUGCGCCUGAGCCUGAAGAAGAGCGUGACCUACCUGGGCAGCGACAUGGAGGUGAGCGGCCUGACCGACAAGAAGAUCAUCGUGAUCCUGCUGGGCUACGCCGGCCUGAUGCUGCUGACCAACAAGAAGAGCCAGGUGAGCUACUACCCCGCCGAGAACAGCUACAGCCGCUGGAGCAAGAAGGGCAGCAAGCGCCUGAUGGCCCUGCAGGAGAAGUACGGCCUGGUGGAGAAGAAGUUCUUCAGCAUCAAGAACAGCUGGGCCAUGGAGACCGGCCGCGAGAACAAGAAGGUGUGGUUCCUGCAGGCCGCCCACAUGUUCUUCAGCAUCAAGAACAGCAAGAAGAUGAACGCCAACACCGUGCGCCUGGAGCCCAGCCUGGGCGAGUACCCCGAGGCCGCCGCCAAGGCCAAGUUCGUGGCCGCCUGGACCCUGAAGGCCGCCGCCGAGGCCGCCGCCAAGACCGGCGCCCUGCUGGCCGCCGGCGCCGCCGCCAUGGCCAAGAGCGGCCUGCGCCAGGACCCCCAGAGCACCGCCGCCGCCACCGUGCUGAAGCGCGCCGUGGAGCUGGACAGCGAGAGCCGCUACCCCCAGGCCCUGGUGUGCUACCAGGAGGGCAUCGACCUGCUGCUGCAGGUGCUGAAGGGCACCAAGGACAACACCAAGCGCUGCAACCUGCGCGAGAAGAUCAGCAAGUACAUGGACCGCGCCGAGAACAUCAAGAAGUACCUGGACCAGGAGAAGGAGGACGGCAAGUACCACAAGCAGAUCAAGAUCGAGGAGAACGCCACCGGCUUCAGCUACGAGAGCCUGUUCCGCGAGUACCUGAACGAGACCGUGACCGAGGUGUGGAUCGAGGACCCCUACAUCCGCCACACCCACCAGCUGUACAACUUCCUGCGCUUCUGCGAGAUGCUGAUCAAGCGCCCCUGCAAGGUGAAGACCAUCCACCUGCUGACCAGCCUGGACGAGGGCAUCGAGCAGGUGCAGCAGAGCCGCGGCCUGCAGGAGAUCGAGGAGAGCCUGCGCAGCCACGGCGUGCUGCUGGAGGUGCAGUACAGCAGCAGCAUCCACGACCGCGAGAUCCGCUUCAACAACGGCUGGAUGAUCAAGAUCGGCCGCGGCCUGGACUACUUCAAGAAGCCCCAGAGCCGCUUCAGCCUGGGCUACUGCGACUUCGACCUGCGCCCCUGCCACGAGACCACCGUGGACAUCUUCCACAAGAAGCACACCAAGAACAUC
