# Supplementary figures and images for: Development of a novel multi-epitope mRNA vaccine candidate to combat SFTSV pandemic
Source: PLoS Negl Trop Dis. 2025 Jan 22;19(1):e0012815. doi: 10.1371/journal.pntd.0012815 (PMC12788908; doi:10.1371/journal.pntd.0012815)

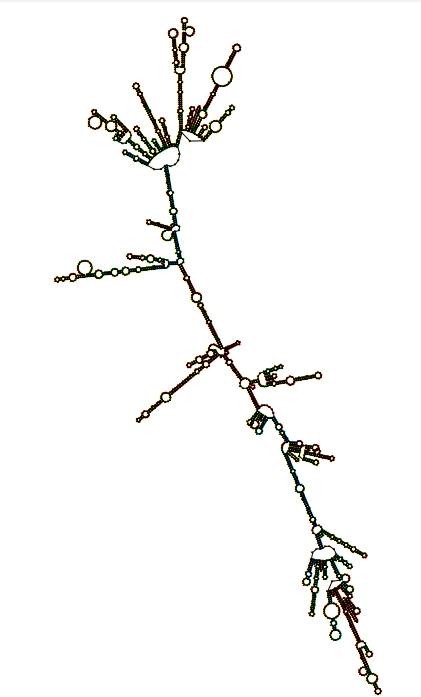

Supplement: S1 Fig — (TIF) [file pntd.0012815.s007.tif]

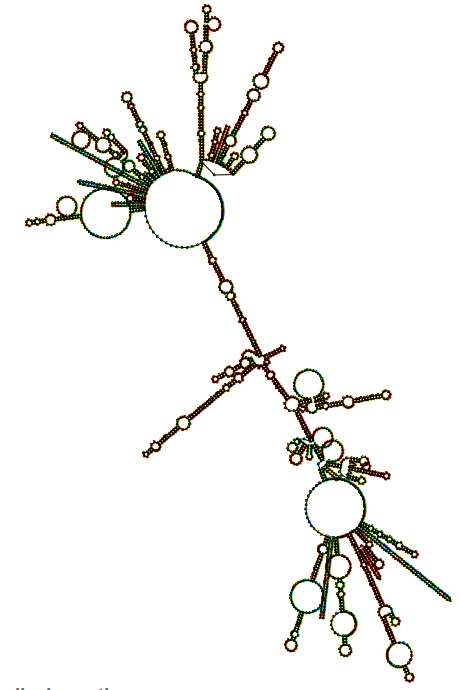

Supplement: S2 Fig — (TIF) [file pntd.0012815.s008.tif]

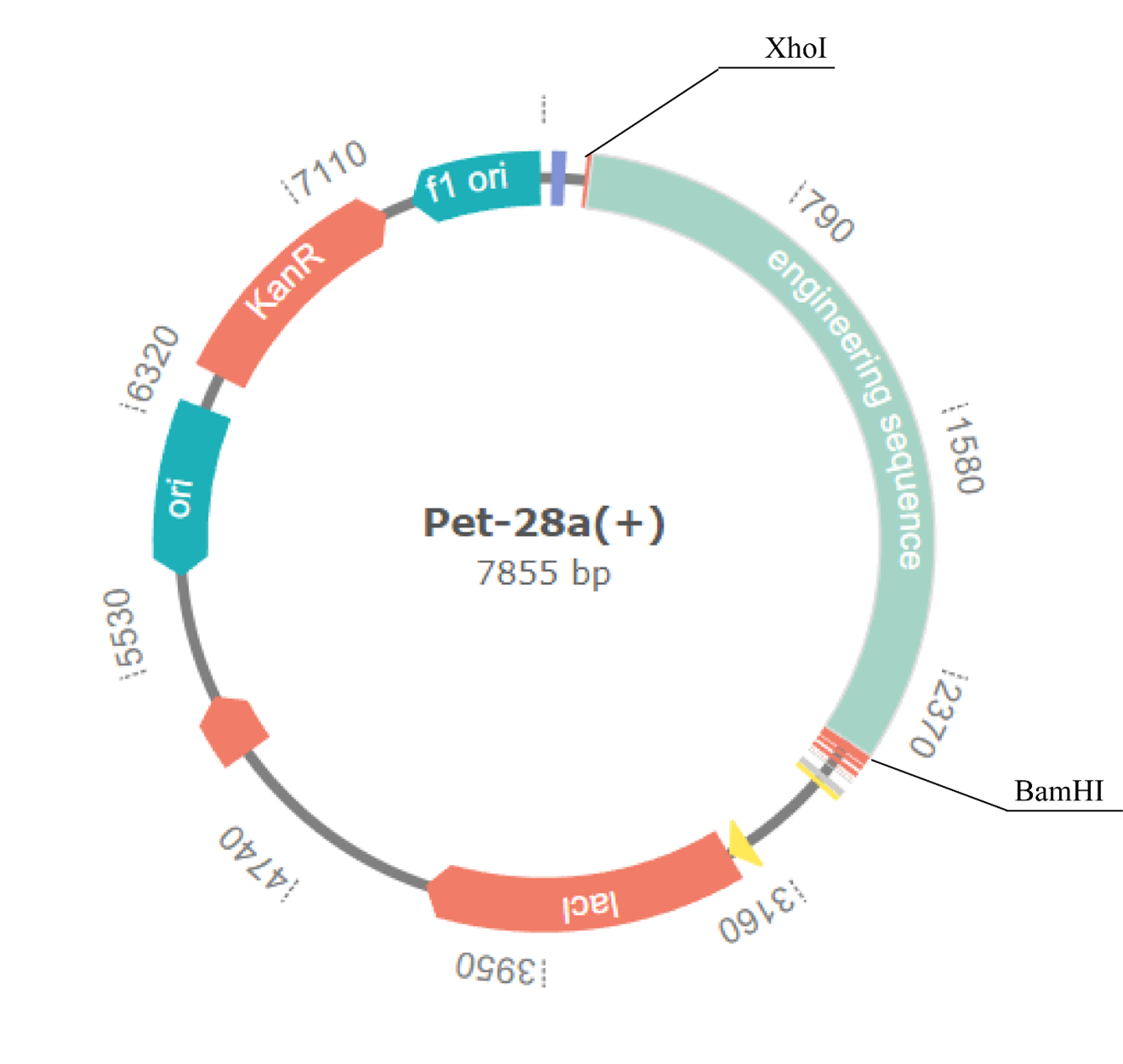

Supplement: S3 Fig — (TIF) [file pntd.0012815.s009.tif]
