# Supplementary material for: Development of a novel multi-epitope mRNA vaccine candidate to combat SFTSV pandemic
Source: PLoS Negl Trop Dis. 2025 Jan 22;19(1):e0012815. doi: 10.1371/journal.pntd.0012815 (PMC12788908; doi:10.1371/journal.pntd.0012815)
Supplement: S1 Table — (DOCX) [file pntd.0012815.s010.docx]

S1 Table. Prediction of mRNA binding with TLRs

|  | RF classifier | \| SVM classifier \|  \| \| --- \| --- \| |
| --- | --- | --- | --- | --- |
| TLR3 | 0.7 | 0.62 |
| TLR7 | 0.7 | 0.44 |
| TLR8 | 0.75 | 0.41 |
| TLR9 | 0.6 | 0.39 |
