# Supplementary material for: Development of a novel multi-epitope mRNA vaccine candidate to combat SFTSV pandemic
Source: PLoS Negl Trop Dis. 2025 Jan 22;19(1):e0012815. doi: 10.1371/journal.pntd.0012815 (PMC12788908; doi:10.1371/journal.pntd.0012815)
Supplement: Attachment — Submitted filename: Review comments.docx [file pntd.0012815.s013.docx]

I would like to thank and appreciate all the authors for conducting a very informative, well-designed, and comprehensive in silico study. This study will undoubtedly be very helpful to the community. Here are some minor comments:

1. The study is impressive and important. The authors have designed this project very well, making it highly informative and valuable for the scientific community.
2. In lines 46-52, it would be better to add citations to support the claims made by the authors about the likelihood of disease exposure and associated problems.
3. The authors have provided sufficient background information, which is very helpful in understanding the importance of this study and why they chose the Gn, Gc, Np, and NSs antigen sequences to construct the vaccine.
4. The detailed screening process to identify the epitope regions is well-designed and impressive. Authors have screened and verified the allergenicity and toxicity of the epitopes, which is important and necessary for studying in silico vaccine safety assessment. However, it is important to note that some viral genomes contain virus-associated molecular patterns (VAMPs) that can bind to intracellular toll-like receptors (TLRs) in the human host. Several studies have reported that intracellular TLR 3, 7, 8, and 9 are responsible for inducing cytokine storms, which can be lethal. Therefore, it would be beneficial to determine whether there are any interactions between intracellular TLRs and the vaccine, and if so, to further analyze these interactions. ((<https://doi.org/10.1002/jmv.26776>)
5. In the immunization simulation study, it would be helpful if the authors also showed the responses of IgM, IgG1, and IgG2, macrophages (MA), and cytokine production.
6. Since immunization simulation assessment is crucial for determining the efficacy of the vaccine, it would be interesting to explore whether other servers, like C-IMMSIM, can be used for immunization simulation studies. If results from different servers are obtained, it might be possible to present all the immunization simulation data as a correlation graph to avoid any bias.

Thank you
