# Supplementary material for: Development of a novel multi-epitope mRNA vaccine candidate to combat SFTSV pandemic
Source: PLoS Negl Trop Dis. 2025 Jan 22;19(1):e0012815. doi: 10.1371/journal.pntd.0012815 (PMC12788908; doi:10.1371/journal.pntd.0012815)
Supplement: Attachment — Submitted filename: response to reviewers.docx [file pntd.0012815.s014.docx]

I would like to thank and appreciate all the authors for conducting a very informative, well-designed, and comprehensive in silico study. This study will undoubtedly be very helpful to the community. Here are some minor comments:

1. The study is impressive and important. The authors have designed this project very well, making it highly informative and valuable for the scientific community.

Thank you for your appreciation.

2. In lines 46-52, it would be better to add citations to support the claims made by the authors about the likelihood of disease exposure and associated problems.

Thanks for your reminder, We have supplemented the references of the data sources.

3. The authors have provided sufficient background information, which is very helpful in understanding the importance of this study and why they chose the Gn, Gc, Np, and NSs antigen sequences to construct the vaccine.

Thanks for your appreciation again.

4. The detailed screening process to identify the epitope regions is well-designed and impressive. Authors have screened and verified the allergenicity and toxicity of the epitopes, which is important and necessary for studying in silico vaccine safety assessment. However, it is important to note that some viral genomes contain virus-associated molecular patterns (VAMPs) that can bind to intracellular toll-like receptors (TLRs) in the human host. Several studies have reported that intracellular TLR 3, 7, 8, and 9 are responsible for inducing cytokine storms, which can be lethal. Therefore, it would be beneficial to determine whether there are any interactions between intracellular TLRs and the vaccine, and if so, to further analyze these interactions. ((<https://doi.org/10.1002/jmv.26776>)

Thank you for your suggestions. We have tested the binding ability of the mRNA vaccine with TLR3, 7, 8, and 9 using the method you recommended. We found that TLR3 may bind to the mRNA vaccine, while TLR7, 8, 9 may not bind to mRNA. Subsequently, we used molecular docking to further analyze the binding between TLR3 and the mRNA vaccine. These contents are now added in line 256-272(methods), line 407-414(results) and line 496-501(discussion).

5. In the immunization simulation study, it would be helpful if the authors also showed the responses of IgM, IgG1, and IgG2, macrophages (MA), and cytokine production.

Thank you for your suggestions. We have reorganized the results of immunological modeling, with the antibody levels displayed in Figure 10A, and the predicted MA levels shown in Figure 10G to replace the repeated predictions of TC cell quantities.

6. Since immunization simulation assessment is crucial for determining the efficacy of the vaccine, it would be interesting to explore whether other servers, like C-IMMSIM, can be used for immunization simulation studies. If results from different servers are obtained, it might be possible to present all the immunization simulation data as a correlation graph to avoid any bias.

This issue has left us somewhat puzzled. In the original study, we conducted immunological modeling using C-IMMSIM and organized the results (Figure 10). As there are currently no other suitable immunological modeling tools, we did not add a second immunological modeling method in the revised manuscript.

Thank you

Major:

1. Some of the bioinformatics tools employed in this study are outdated, potentially compromising the accuracy of the results. Specifically, for predicting CTL epitopes, the authors utilized NetCTL 1.2, a method introduced in 2007. To enhance accuracy and reliability, it is recommended to use more advanced tools like NetMHCpan-4.1 or MixMHCpred-3.0. The authors should consider validating their proposed vaccine using these state-of-the-art tools to substantiate their findings.

Thanks for your reminder, NetMHCpan4.1 validates the affinity between CTL and MHC molecules. NetMHCIIpan 4.3 validates the affinity between HTL and MHC II molecules. The selected epitopes exhibit strong binding capability with MHC molecules. These results can be found in supplement materials named as epitope validation.

2. There is insufficient evidence to validate the claim regarding autoimmunity. In the discussion, the authors stated, "Moreover, no homology was shown between selected epitopes and human or human gut commensal bacteria, to avoid inducing latent autoimmunity damage." However, the tools used to calculate this homology were not specified. Additionally, the threshold for identity that distinguishes homology from non-homology was not mentioned. The authors are encouraged to provide further details on the methodologies and criteria used for these homology assessments.

Thank you for your suggestions. We have provided specific homology testing methods in lines 158-165, reorganized various original data and results used, and added them to the supplementary materials, named as “Homology Testing of Intestinal Microbiota.”

Minor

1. The caption of Table 4, currently labeled as 'formation B epitopes', should be revised to 'conformational B-cell epitopes' for clarity and accuracy.

Thank you for your reminder, we have corrected this typographical error.

2. In the 'Epitope screening' part of the abstract figure, it appears that the authors use MHC-peptide binding process to represent the epitopes. However, CTL should correspond to MHCI, which is depicted with a single leg (alpha3), and HTL should correspond to MHCII, which is depicted with two legs (alpha2 and beta2). Therefore, the two diagrams should be swapped for accuracy.

Thanks a lot, this was a graphical error, and we have addressed this issue.

3. Line 291: 'epitopes in the structure(Table 4), the overall score varied from '0.855-0.528'. Please consider revise it to 'The overall score varied from 0.528 to 0.855.'

Thank you for carefully reviewing our manuscript, and this error has been corrected.
